# Supplementary material for: Affinity maturation of antibody responses is mediated by differential plasma cell proliferation
Source: Science. Author manuscript; Available in PMC 2025 Mar 26. (PMC11938350; doi:10.1126/science.adr6896)
Supplement: 1 [file NIHMS2063056-supplement-1.pdf]

Science
AAAS

Supplementary Materials for

Affinity maturation of antibody responses is mediated by differential plasma cell proliferation

Andrew J. MacLean[1,*], Lachlan P. Deimel[1], Pengcheng Zhou[1], Mohamed A. ElTanbouly[1], Julia Merkenschlager[1], Victor Ramos[1], Gabriela S. Santos[1], Thomas Hägglöf[1], Christian T. Mayer[2], Brianna Hernandez[1], Anna Gazumyan[1], Michel C. Nussenzweig[1,3,*]

*Corresponding authors: nussen@rockefeller.edu, amaclean@rockefeller.edu

The PDF file includes:

Materials and Methods
Figs. S1 to S8
References 40-50

Materials and Methods

Mice

S1pr2-CreERT2 mice were kindly provided by T. Kurosaki (*21*). B1-8^{hi}, mCherry and DEC^{ko} were as described (*13, 25, 28*). $R26^{lsl-ZSGreen}$ and C57Bl/6J mice were purchased from Jackson Laboratories. $R26^{INDIA/INDIA}$ were kindly provided by C. Mayer (*24*). Blimp1-CreERT2 mice were generated by insertion of a tamoxifen inducible Cre into the 3' untranslated region of the Prdm1 (Blimp1) gene (Blimp1-CreERT2) linked by a P2A sequence. To verify the specificity of the Blimp1-CreERT2 driver we combined it with the $R26^{lsl-ZSGreen}$ indicator allele. Male mice aged 6-12 weeks were used in all experiments. Animals were housed at The Rockefeller University Comparative Bioscience Center, and all animal procedures were performed following protocols approved by the Rockefeller University Institutional Animal Care and Use Committee. Animals were housed at an ambient temperature of 22C and a humidity of 30–70% under a 12h–12h light–dark cycle with ad libitum access to food and water. For experimental endpoints, mice were euthanized by cervical dislocation.

Immunizations and tamoxifen treatment

NP-OVA immunizations were performed by subcutaneous footpad injection of 12.5ug NP_{16}-OVA (Biosearch Technologies) in 33% alhydrogel (Invivogen). Recombinant SARS-CoV2 RBD was produced as described (*40*). For RBD immunization, 5ug RBD was administered per footpad, in 33% alhydrogel. 50ul of Tenivac® (tetanus and diphtheria toxoids, adsorbed; Sanofi Pasteur) was administered per footpad. For tamoxifen administration, each mouse received one dose of 12mg tamoxifen (Sigma-Aldrich, T5648) prepared in corn oil by oral gavage at the indicated timepoint.

Antibody infusion and FTY720 administration

For GC experiments 500ug of anti-CD40L (Clone MR1; BioXCell) was administered intravenously on D10 and 100ug on D12 after immunization. For longitudinal serological experiments 100ug anti-CD40L was also given on D16, D20 and D24 to maintain depletion.
For partial IL-21R blocking, 50ug of anti-IL-21R (Clone 4A9, BioXCell), was administered subcutaneously on D10.
For PC depletion, TACI-Ig was produced as described (*41*), with the following differences. Briefly, an expression vector for TACI-Ig fusion protein was generated by conjoining DNA sequences encoding the pre-pro signal sequence from human tissue plasminogen activator, the extracellular domain of mouse TACI (aa2–82), and a mutated H chain Fc region from the C57BL/6 mouse IgG2c (lacking CH1). The L235E, E318A, K320A, and K322A aa substitutions were introduced based on codon usage into BL/6 IgG2c Fc as detailed (*41*). TACI-Ig was produced in Expi293F (Thermo Fisher Scientific). 100ug TACI-Ig was administered intravenously on D10, D12, D16, D20 and D24.

To prevent PC egress from LNs, 1mg/kg FTY720 (Selleck Chemicals) was administered intravenously on D10 and D14 after immunization as indicated.
Anti-DEC205-CS and anti-DEC205-OVA were produced as described (*42*). Mixtures of anti-DEC205-OVA and -CS were administered at the indicated ratios, with a total of 5ug administered to each footpad, on D6 after immunization. For DEC experiments, a cell transfer approach was utilized to allow targeted antigen delivery to a fraction of GC B cells (90% DECko/10%DECwt B cells). Previous experiments in our lab have shown this approach to maintain the competitive

nature of the GC reaction, and allow the help to be focused on a small proportion of responding cells (*28*).

Bait preparation
Avi-tagged recombinant SARS-CoV2 Wuhan Hu-1 RBD (*40*) was biotinylated using the Avidity BirA500 biotinylation kit (Avidity, EC 6.3.4.15) following the manufacturer's instructions. rTTHC (Fina Biosolutions) and CRM197 (Fina Biosolutions) were biotinylated using Pierce EZ-Link biotinylation kit (Thermo Scientific, 31497). Excess biotin was removed by diafiltration with 100kDa cutoff. A 5-fold molar excess of biotin was used for the reaction.

Flow cytometry
Briefly, LNs were collected into 1.5ml Eppendorf tubes and dissociated using a pestle. Single cell suspensions were incubated in Fc block (BD Biosciences) for 15min, followed by primary antibody staining at 4°C for 30min. For intracellular staining of Ki67, cells were permeabilized using BD Cytofix/Cytoperm kit, washed in the supplied permeabilization buffer and stained for 30min at 4°C.

Bait staining was performed with a combination of two different fluorescently-labelled streptavidins per bait. Biotinylated antigens were individually pre-incubated with each streptavidin-fluorophore before staining to allow tetramer formation, then cell suspensions were stained for 30min on ice, before staining for other surface markers as outlined above.

Antibodies used were as follows: Anti-mouse FC block (2.4G2, BD, 553142), Anti-mouse CD4 (RM4-5, Invitrogen, 47-0042-82), Anti-mouse CD95 (Jo2, BD, 557653), Anti-mouse CD38 (90, ThermoFisher, 56-0381-82), Anti-mouse B220 (RA3-6B2, BD, 748867), Anti-mouse CD138 (218-2, Biolegend, 142518), Anti-mouse TACI (8F10, BD, 742840), Anti-mouse CD86 (GL1, BD, 740688), Anti-mouse CXCR4 (2B11/CXCR4, BD, 558644), Anti-mouse CD45.1 (A20, Biolegend, 110708), Anti-mouse CD45.2 (104, Biolegend, 109839), Anti-mouse Ki67 (B56, BD, 561126), Anti-mouse TCRb (H57-597, Invitrogen, 47-5961-82).

Cell sorting and VDJ Seq analysis
Single mouse GC B cells or PCs were purified and processed as described (*43*). Briefly, samples were single-cell sorted into 96-well plates containing 5ul lysis buffer (TCL buffer (Qiagen, 1031576); 1% 2-mercaptoethanol). Single cell Ig sequencing was performed using the nested PCR protocol (*43*). For IgL sequencing the following primers were used:
PCR1: 1mFL1 (ACTTATACTCTCTCCTGGCTCTC), 1mFL2 (CTCTTCTTCTTCTTTGTTCTTCATTGCT), 1mRL (GTACCATYTGCCTTCCAGKCCACT). Annealing temp=46°C. PCR2: 2mFL1 (CAGGCTGTTGTGACTCAG), 2mFL2 (CAACTTGTGCTCACTCAG), 2mRL (CTCYTCAGRGGAAGGTGGRAACA). Annealing temp=57°C. The 2mRL primer was used for sequencing. PCR products were Sanger sequenced and analyzed using Geneious Prime, and our Ig analysis bioinformatic pipeline (*44-46*). All scripts and data used to process antibody sequences are publicly available on GitHub at https://github.com/stratust/igpipeline/tree/igpipeline2_timepoint_v2. IgL, IgK and IgG/IgM IgH chains were paired (1xIgH, 1xIgL only) and analyzed.

qPCR

200-500 cells were sorted into lysis buffer supplied with the SuperScriptIV Single Cell/Low Input cDNA preamp kit (Thermo Scientific, 11752048). Samples were treated as per kit instructions, and 13x cycles of preamplification were performed. Taqman probes for GAPDH(Mm99999915_g1), HPRT(Mm00446968_m1), Nr4a1(Mm01300401_m1), Irf4(Mm00516431_m1), Myc(Mm00487804_m1), Tfap4(Mm00473137_m1) were used for this assay, in conjunction with Taqman fast advanced master mix (Applied Biosystems, 4444556). For data presentation, relative expression was calculated normalized to GAPDH.

ELISA

For detection of NP-reactive serum IgG, 96-half-well plates (Corning, 3690) were coated with 25ul NP_7-BSA or NP_{28}-BSA (Biosearch Technologies) at 10ug/ml in PBS overnight at 4°C. Plates were washed 2x with washing buffer (PBS, 0.05% Tween20, Sigma-Aldrich) and blocked in 150ul PBS, 2% BSA for 1h at RT. Plates were washed 4x more, and incubated for 2h at room temperature with serially diluted serum samples. B1-8^{hi} IgG was used as a standard on each plate to calculate absolute concentrations of NP_7- and NP_{28}- reactive IgG in serum. After 4 more washes, secondary HRP-conjugated anti-mouse IgG (Jackson, 115-035-071; 1:5000 dilution) was added for 1h at room temperature. Plates were washed 6x and developed using 3,3',5,5'-tetramethylbenzidine (Thermo Scientific) for 3min, followed by addition of H_2SO_4. 450nm absorbance values were immediately measured using a microplate reader (Fluostar Omega, BMG Labtech).

Confocal microscopy and analysis

Lymph nodes were prepared for confocal imaging as previously described (*47*). Briefly, LNs were collected from mice and fixed in 4% PFA for 2h. LNs were washed in PBS, immersed in 30% sucrose for cryoprotection for 12h, and embedded in OCT (Optimal cutting temperature compound; TissueTek) blocks. Tissues were sectioned at 7um on a cryostat (Microm) and blocked with mouse serum (Rockland) and Fc block for 30min. Slides were incubated at RT with the indicated antibodies for 6h, washed in PBS and mounted using fluoromount-G (ThermoScientific). Microscopy was performed on an inverted laser scanning microscope LSM980 (Zeiss).

For quantification, the Spots function in Imaris imaging software (Bitplane) was used. $CD138^+$ PC were identified based on CD138-PE fluorescence intensity, cell quality (a metric which is automatically quantified in the Spots function), and these cells were sub-grouped based on staining intensity of NP and/or Ki67. All spots were manually verified.

Single-cell libraries processing

For single-cell B cell receptor sequence reconstruction we used Cell Ranger (v.7.1.0) with mouse reference genome GRCm38. Hashtag-oligos (HTOs) UMI counts were processed using CITE-Seq-Count (v.1.4.5). We utilized MULTIseqDemux from Seurat (v4.1.1) to demultiplex, visualize and categorize cells as either GC or PC, based on the measured surface expression of CD138, CD95, CCR6 and CD86 by antibody-conjugated oligos. A small fraction of ZSG^+ cells showing expression of CCR6 but lacking CD95 were included, but these cells were not classified as GC or PC, and were excluded from subsequent analysis.

Computational analyses of antibody sequences

Single-cells heavy and light chains were paired using in-house R scripts and subsequently analyzed using igpipeline v2.0 (https://github.com/stratust/igpipeline/tree/igpipeline2_timepoint_v2), as previously described (*44*), using the mouse IMGT database (*48*) as reference. The

paired IgH and IgL chains of antibodies from the same clonal progeny were merged and aligned to the mouse IMGT germline (GL) sequence using mafft v7.520 (*49*) with default parameters, except for –globalpair. Genotype-collapsed phylogenetic trees (GCtrees) of clonal lineages were inferred using GCTree v4.1.2 (https:// github.com/matsengrp/gctree) (*50*). Each node represents a unique IgH and IgL combination with node size indicating the number of identical sequences. The small dotted nodes represent the unobserved ancestral. A CITE-Seq library including CD138, CD95, CCR6 and CD86 was used to assign GC or PC-identity to cell barcodes, and for UMAP generation of analyzed populations.

Statistical analysis

Details of statistics including tests used, exact values and n numbers are indicated in figure legends and/or main text. Quantification and statistical analyses were performed in GraphPad Prism (Version 10.2.3), unless otherwise detailed in this methods section. Graphs generated using Prism were assembled into figures using Adobe Illustrator. Flow cytometry analysis was performed in FlowJo v.10.10.0 software (BD).

Supplementary Figure 1.

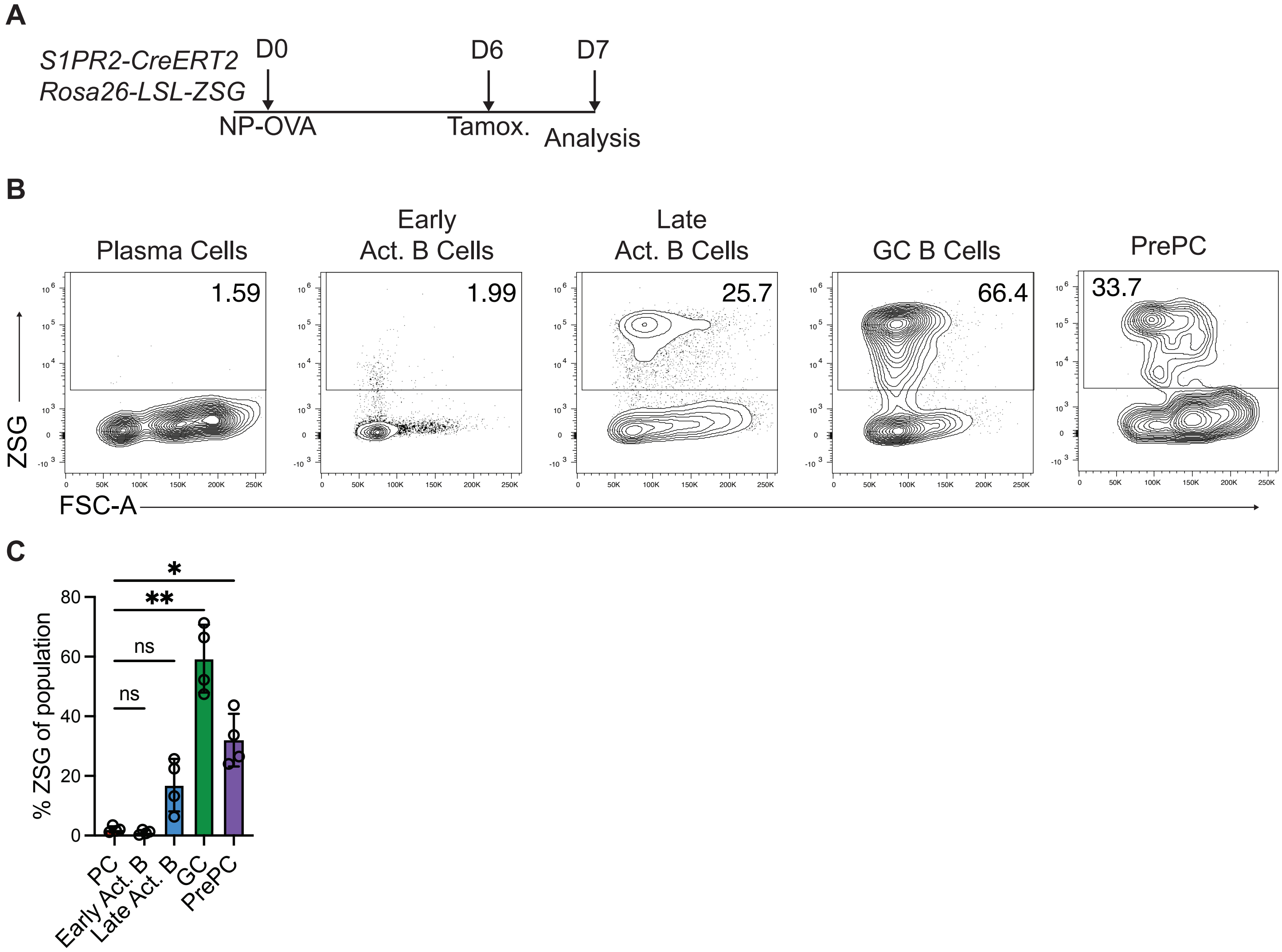

Supplementary Fig. 1. S1PR2-CreERT2 labels GC B cells and late activated B but not mature plasma cells, related to fig.1.

(**A**) Experimental outline. (**B**) Representative flow cytometry plots showing ZSG expression in PCs (TACI$^+$ CD138$^+$), early activated B cells (TACI$^-$ CD138$^-$ B220$^+$ CD38$^+$ GL7$^+$ Fas$^-$), late activated B cells (TACI$^-$ CD138$^-$ B220$^+$ CD38$^+$ GL7$^+$ Fas$^+$), GC B cells (TACI$^-$ CD138$^-$ B220$^+$ CD38$^-$ GL7$^+$ Fas$^+$) and PrePC (TACI$^-$ B220$^+$ CD38$^-$ GL7$^+$ Fas$^+$ CD138$^+$). (**C**) Quantitation of data presented in B. Each point represents one mouse. Data in B-C represent one of three experiments performed. ns, not significant, ** $P<0.005$; ordinary one-way ANOVA.

Supplementary Figure 2.

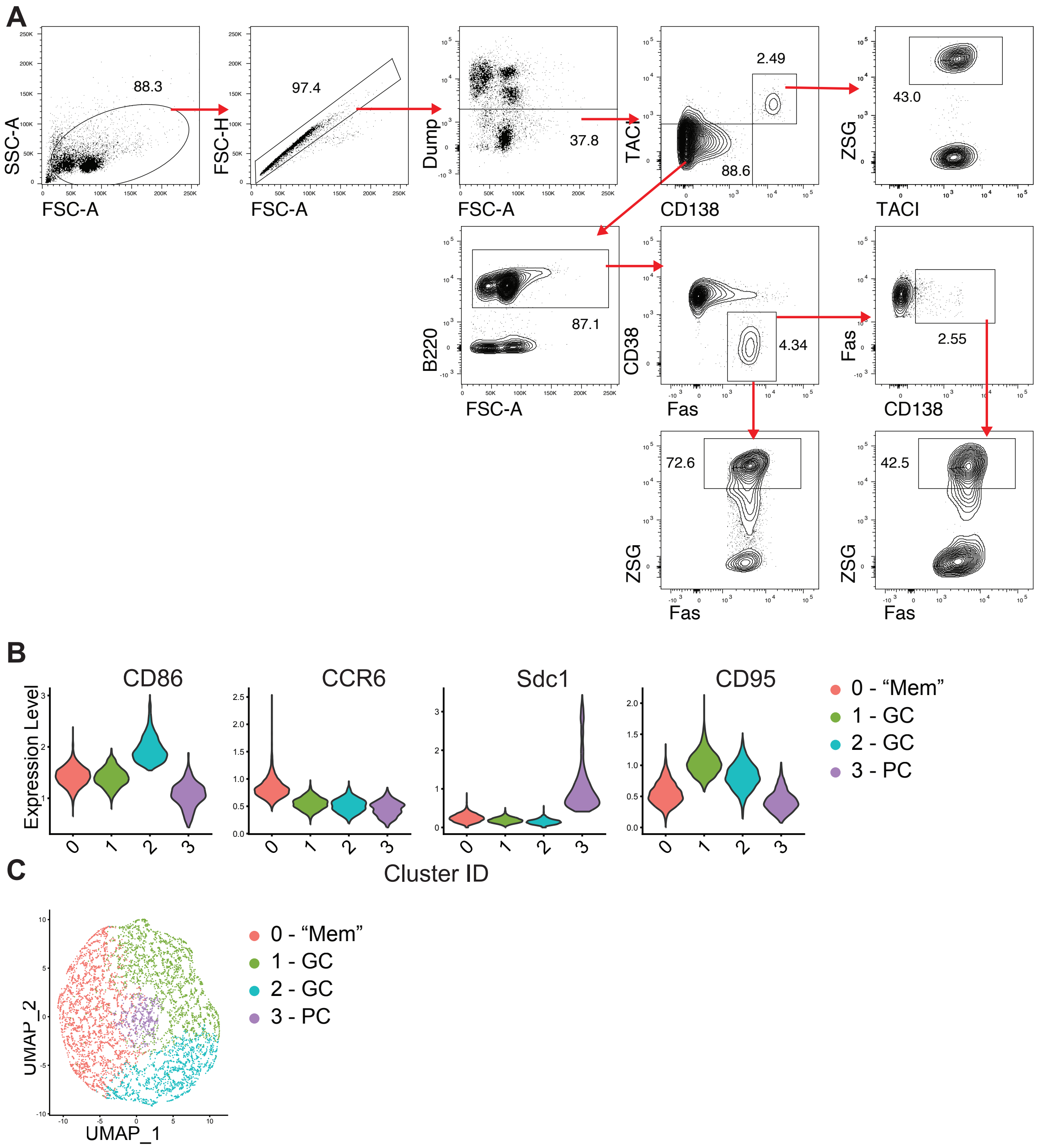

Supplementary Fig. 2. Identification of GC B and PC, related to fig.1

(**A**) Gating strategy for ZSG^{+} PCs ($Dump^{-}$ $CD138^{+}$ $TACI^{+}$), GC B ($Dump^{-}$ $TACI^{-}$ $B220^{+}$ $CD38^{lo}$ Fas^{+}) and prePCs ($Dump^{-}$ $TACI^{-}$ $B220^{+}$ $CD38^{lo}$ Fas^{+} $CD138^{+}$), from animals treated as in Fig. 1A. Gating approach depicted was used for these populations throughout unless otherwise stated. (**B**) Expression levels of CITE-seq surface staining in clusters identified as PC and GC in two separate sequencing runs. (**C**) Uniform manifold approximation and projection (UMAP) of the above data. Data are pooled from 5 mice, and represent two experiments performed.

Supplementary Figure 3.

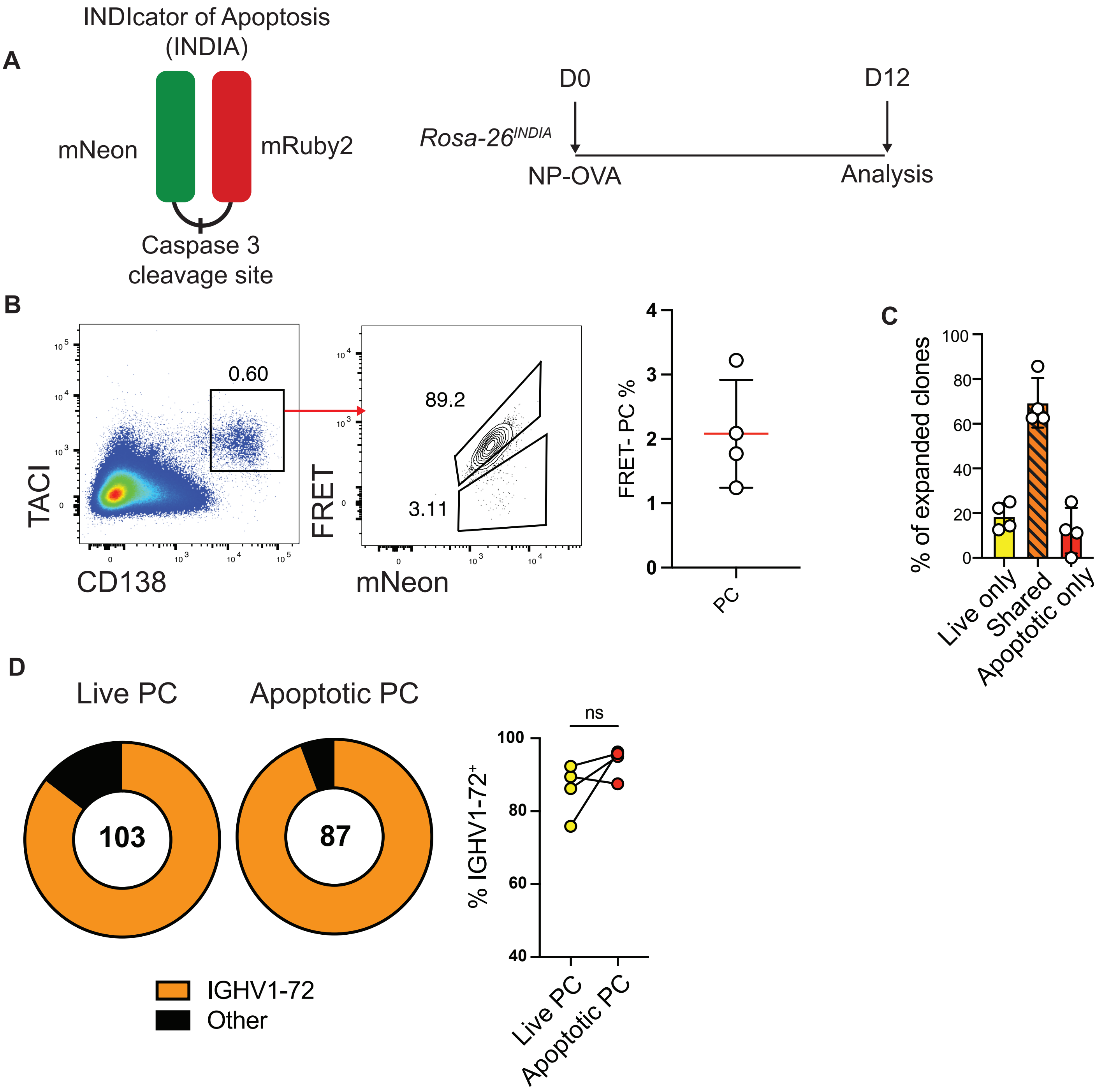

Supplementary Fig. 3. Plasma cell apoptosis is not associated with BCR affinity.

(**A**) Left, Diagram of INDIA reporter. Right, Experimental outline for (**B-D**). (**B**) Left, representative flow cytometry plot showing gating for CD138^{+}TACI^{+} PCs, FRET (BB630 channel) and mNeon. Right, Quantitation of FRET^{+} PCs. (**C**) Frequency of clones found only among live PCs, only in FRET^{-} apoptotic PCs or 'shared' clones found in both populations. Each point represents one mouse. (**D**) Left, frequency of FRET^{+} live PCs or FRET^{-} apoptotic PCs expressing IGHV1-72 antibodies. Right, summary of IGHV1-72 frequencies, each point represents one mouse. All experiments were performed at least twice.

Supplementary Figure 4.

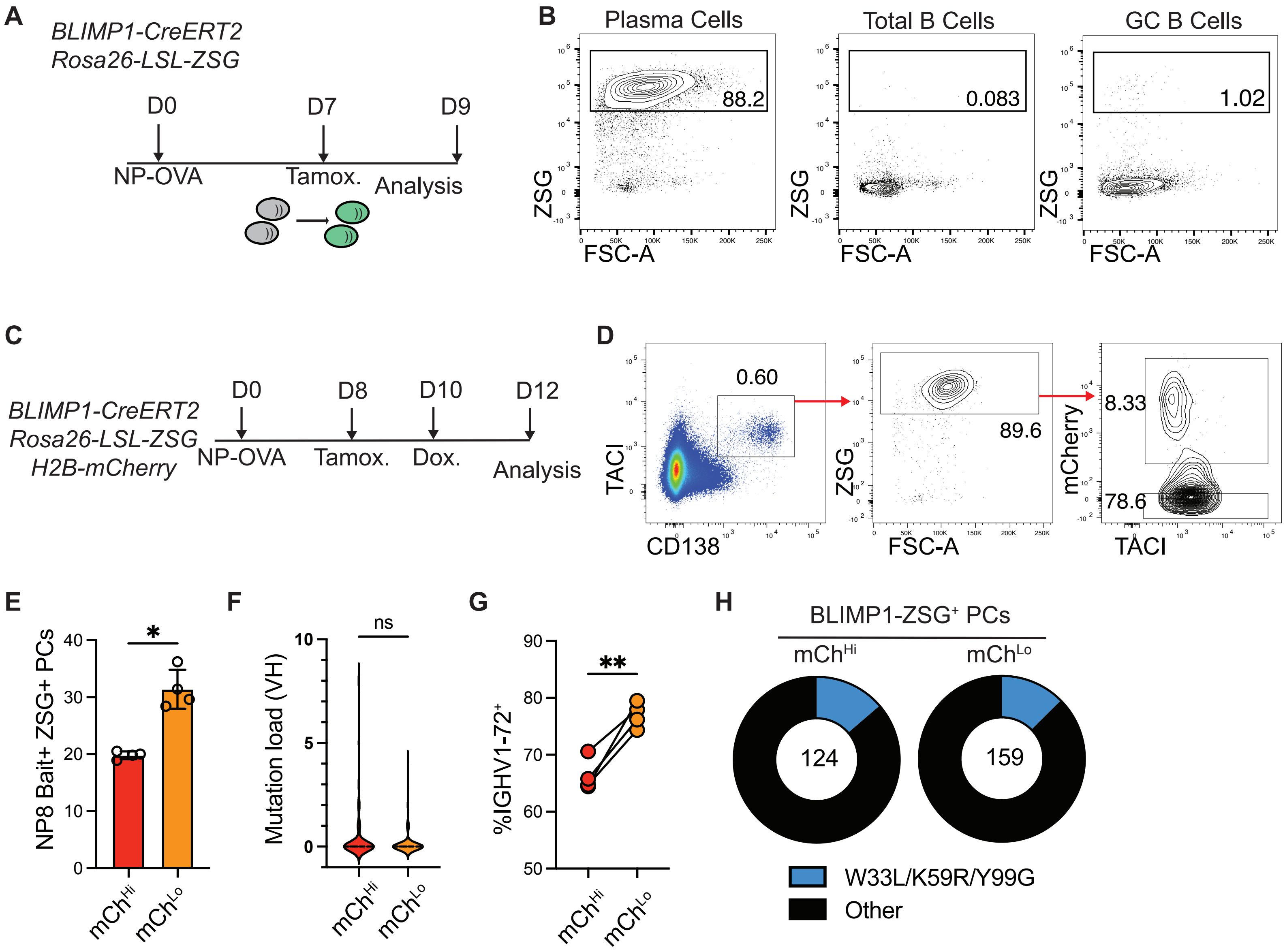

Supplementary Fig. 4. Proliferating PCs are enriched among high-affinity antigen binding cells. (**A**) Experimental layout for **B**. (**B**) Representative flow cytometry plots showing Blimp1-CreERT2-driven fate mapping of PCs ($CD138^+TACI^+$), total B cells ($TACI^-$ $CD138^-$ $B220^+$) and GC B cells ($TACI^-$ $CD138^-$ $B220^+CD38^-Fas^+$). (**C**) Experimental layout for **D-H**. (**D**) Flow cytometry profile showing $TACI^+CD138^+ZSG^+$ PCs and gating for mCh^{hi} and mCh^{lo} cells from pLNs. (**E**) Quantitation of NP bait staining frequency among mCh^{hi} and mCh^{lo} PCs on D12 after immunization. (**F**) Number of VH mutations in ZSG^+ PC populations.(**G**) Frequency of mCh^{Hi} or mCh^{Lo} ZSG^+ PCs expressing IGHV1-72. (**H**) Frequency of high affinity mutation containing sequences among IGHV1-72^+ expressing mCh^{Hi} or mCh^{Lo} ZSG^+ PCs. ns, not significant * $p<0.05$, ** $p<0.005$. (**E, G**) paired two-tailed Student's t-test; (**F**) unpaired Student's t-test. Data are pooled from 2 independent experiments, n=4.

Supplementary Figure 5.

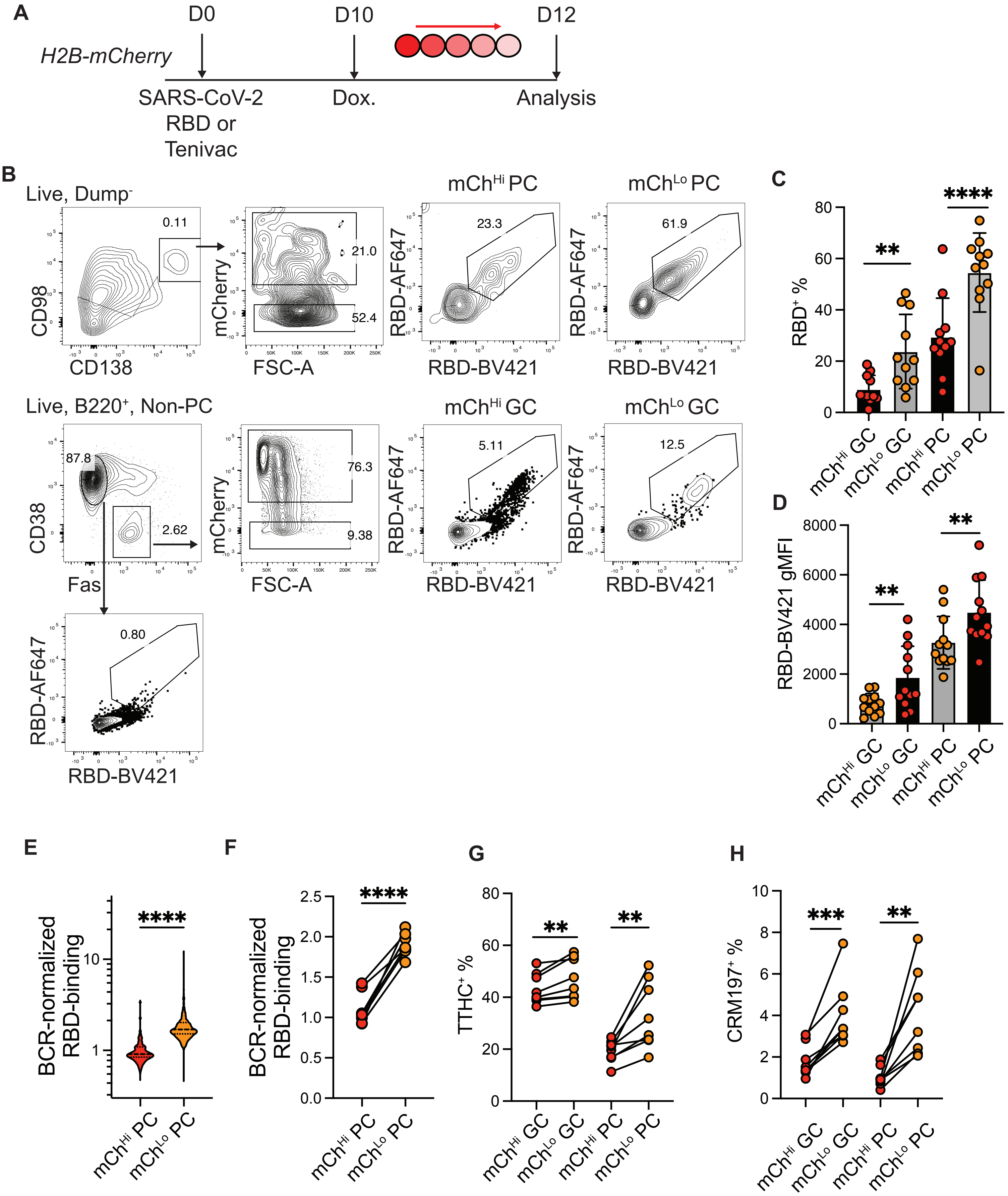

Supplementary Fig. 5. Proliferating PCs are enriched among high-affinity antigen binding cells. (**A**) Experimental layout used in (**B-D**). (**B**) Representative flow cytometry plots showing gating on mCh^{hi} and mCh^{lo} $CD98^{+}CD138^{+}$ PCs, and $mCh^{hi/lo}$ $B220^{+}CD38^{-}Fas^{+}$ GC B cells. Right panels display representative dual antigen bait staining for Sars-CoV-2 RBD. Naïve $B220^{+}CD38^{+}Fas^{-}$ B cells were used as a negative control for bait staining. (**C**) Quantitation of RBD staining frequency among mCh^{hi} and mCh^{lo} GC B cells and PCs on D12 after SARS-CoV-2 RBD immunization. (**D**) Geometric mean fluorescence intensity (gMFI) of RBD-BV421 staining among mCh^{hi} and mCh^{lo} PC and GC B. (**E**) Violin plots displaying cellular distribution of BCR-normalized bait binding. (**F**) Average BCR-normalized bait binding. Each point represents one mouse. (**G-H**) Quantitation of tetanus toxoid heavy chain fragment c (TTHC; **E**) and detoxified diphtheria toxin (CRM197; **F**) bait binding on D12 after Tenivac immunization. * $p<0.05$, ** $p<0.005$, ***$p<0.0005$, **** $p<0.0001$. (**C-D, F-H**) Paired two-tailed Student's t-tests. (**E**), Mann-Whitney test. Data in B-F and G-H are each representative of 3 independent experiments.

Supplementary Figure 6.

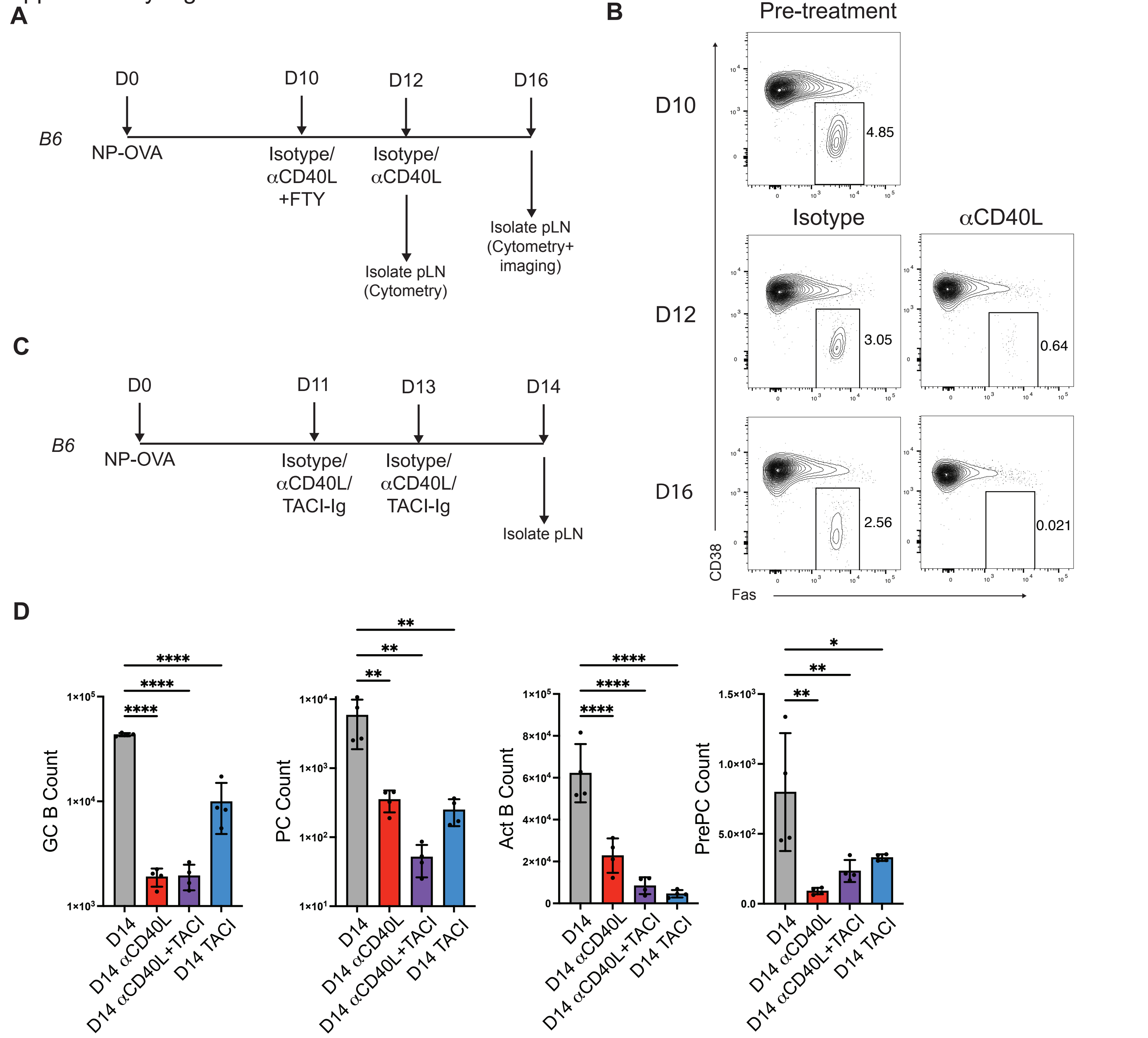

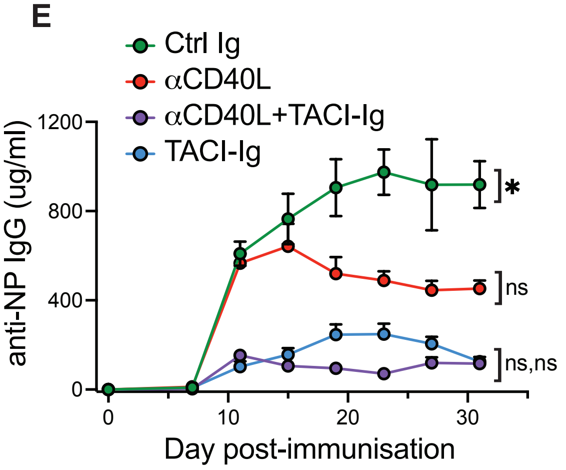

F

Comparison	Treatment group			
	Ctrl (Isotype)	αCD40L	TACI-Ig	αCD40L+ TACI-Ig
D15 vs D11	ns	ns	ns	ns
D19 vs D11	**	***	ns	ns
D23 vs D11	****	**	ns	ns
D27 vs D11	****	**	ns	ns
D32 vs D11	***	*	ns	ns

Supplementary Fig. 6, related to Fig. 3 and Fig.4. GC B and PC depletion kinetics.

(**A**) Experimental layout used in (**B**). (**B**) Representative cytometry plots showing GC B cell depletion in pLNs on D12 and D16 after immunization. (**C)** Experimental layout used in (**D**). (**D**) Quantitation of GC B, PC, activated B and prePCs after treatment with depleting antibodies as described in (**C)**. (**E**) Total serum NP-binding IgG, from mice treated as in Fig. 4H, as measured by NP_{28}-binding. Statistical comparisons shown represent results of a mixed-effects analysis, from endpoint D32 vs D11 onset of treatment. (**F**) Results of mixed effects analysis comparing affinity maturation (NP_7/NP_{28} ratio) of the specified timepoints vs D11, in the same group. Data are presented in Fig.4D,E. * p<0.05, ** p<0.005, ***p<0.0005, **** p<0.0001, ns not significant. (**D**) Ordinary one-way ANOVA (all plots); (**E,F**) mixed-effects analysis.

Supplementary Figure 7.

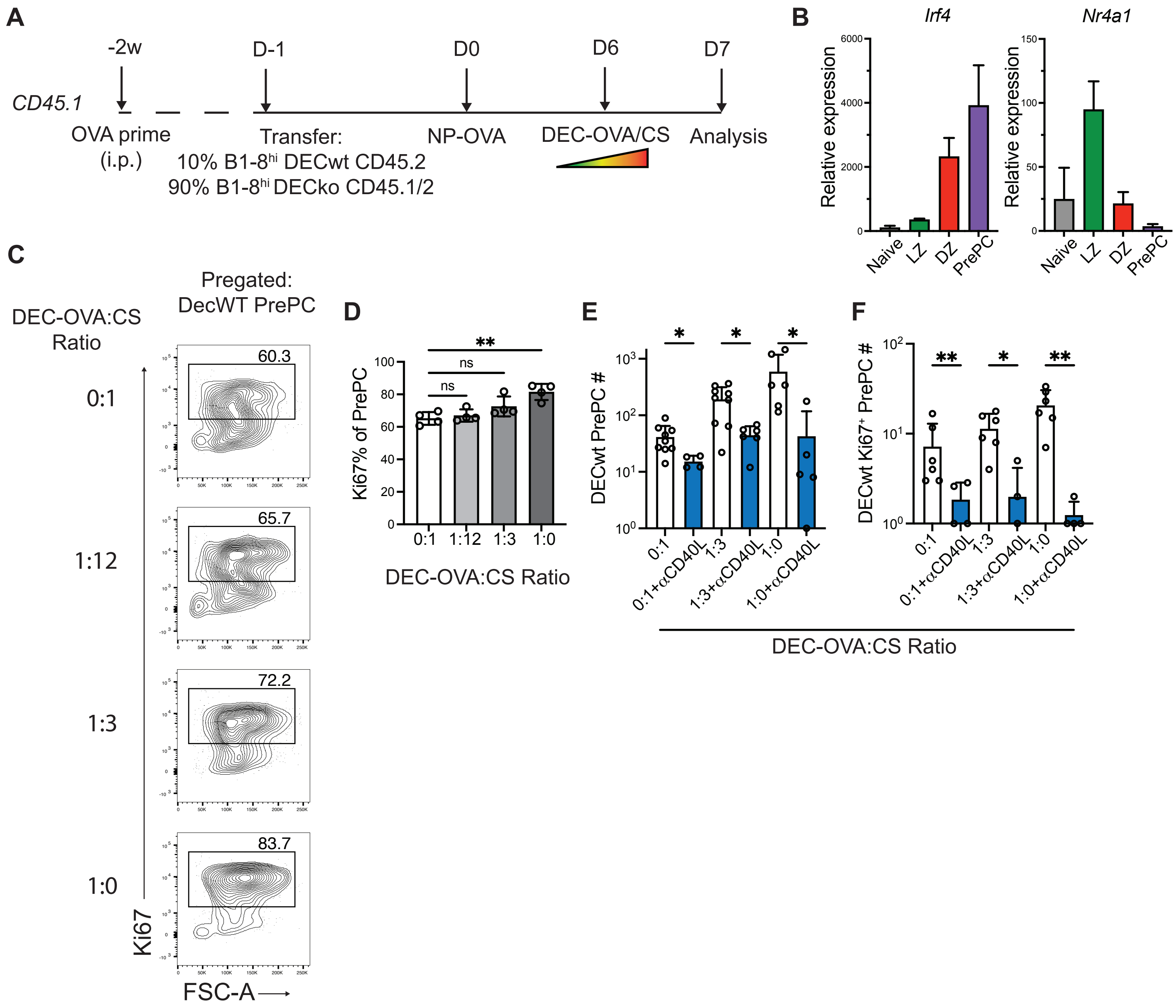

Supplementary Fig 7. PrePC response to T cell help.

(**A**) Experimental layout used in (**B-D**). (**B**) qPCR of purified naïve B (grey bars) LZ B (green bars), DZ B (red bars) and prePC (purple bars) showing GAPDH-normalized relative expression for *Irf4* (left) and *Nr4a1* (right). (**C**) Representative cytometry plots showing frequency of Ki67 staining among prePC, after DEC-OVA:DEC-CS administration. (**D**) Percentage of $Ki67^+$ cells among total DEC^{WT} prePC. (**E-F**) Quantitation of DEC^{WT} prePCs (**E**) and $Ki67^+$ DEC^{WT} prePCs (**F**) 72h after anti-DEC administration, with or without aCD40L treatment as indicated (also see Fig.5G). * $p<0.05$, ** $p<0.005$. (**D-F**) Kruskal-Wallis tests.

Supplementary Figure 8.

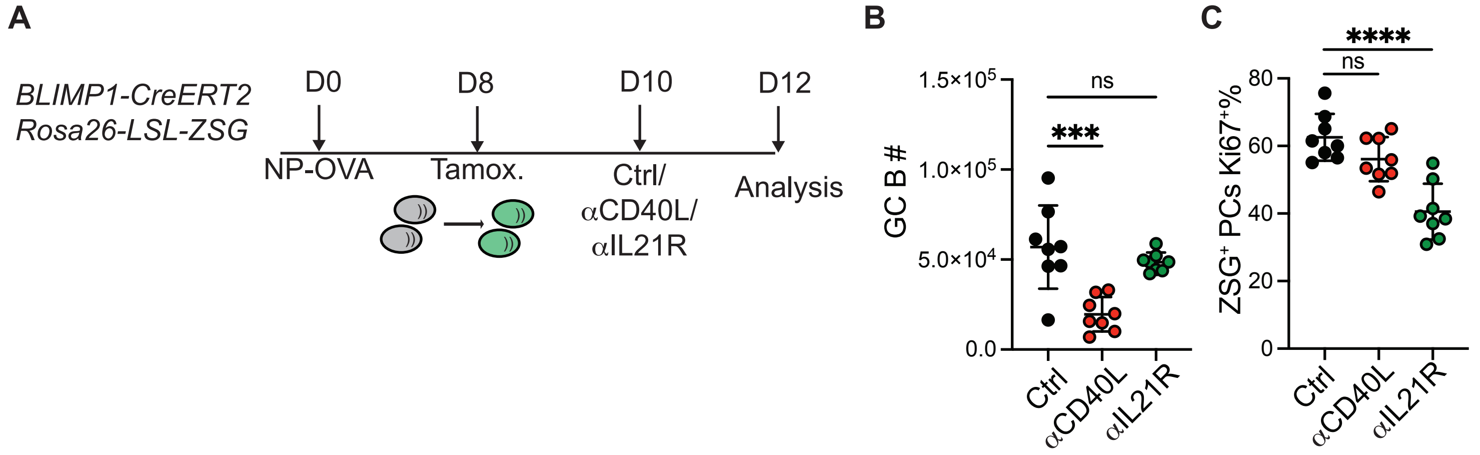

Supplementary Fig 8. IL-21R supports post-GC expansion of PCs. (**A**) Experimental layout. (**B**) GC B cell numbers from mice treated with aCD40L, aIL-21R or isotype control antibodies between D10-D12. (**C**) Frequency of Ki67+ dividing cells amongst $CD138^+$ $TACI^+$ ZSG^+ fate-mapped PCs. ns, not significant ** p<0.005; (**B**,**C**) Ordinary one-way ANOVA. Data are pooled from 3 independent experiments.
